# Identifying appropriate prediction models for estimating hourly temperature over diverse agro-ecological regions of India

**DOI:** 10.1038/s41598-023-34194-9

**Published:** 2023-05-13

**Authors:** Santanu Kumar Bal, V. P. Pramod, V. M. Sandeep, N. Manikandan, M. A. Sarath Chandran, A. V. M. Subba Rao, P. Vijaya Kumar, M. Vanaja, V. K. Singh

**Affiliations:** ICAR-Central Research Institute for Dryland Agriculture, Hyderabad, Telangana 500059 India

**Keywords:** Climate sciences, Agroecology

## Abstract

The present study tests the accuracy of four models in estimating the hourly air temperatures in different agroecological regions of the country during two major crop seasons, *kharif* and *rabi*, by taking daily maximum and minimum temperatures as input. These methods that are being used in different crop growth simulation models were selected from the literature. To adjust the biases of estimated hourly temperature, three bias correction methods (Linear regression, Linear scaling and Quantile mapping) were used. When compared with the observed data, the estimated hourly temperature, after bias correction, is reasonably close to the observed during both *kharif* and *rabi* seasons. The bias-corrected Soygro model exhibited its good performance at 14 locations, followed by the WAVE model and Temperature models at 8 and 6 locations, respectively during the *kharif* season. In the case of *rabi* season, the bias-corrected Temperature model appears to be accurate at more locations (21), followed by WAVE and Soygro models at 4 and 2 locations, respectively. The pooled data analysis showed the least error between estimated (uncorrected and bias-corrected) and observed hourly temperature from 04 to 08 h during *kharif* season while it was 03 to 08 h during the *rabi* season. The results of the present study indicated that Soygro and Temperature models estimated hourly temperature with better accuracy at a majority of the locations situated in the agroecological regions representing different climates and soil types. Though the WAVE model worked well at some of the locations, estimation by the PL model was not up to the mark in both *kharif* and *rabi* seasons. Hence, Soygro and Temperature models can be used to estimate hourly temperature data during both *kharif* and *rabi* seasons, after the bias correction by the Linear Regression method. We believe that the application of the study would facilitate the usage of hourly temperature data instead of daily data which in turn improves the precision in predicting phenological events and bud dormancy breaks, chilling hour requirement etc.

## Introduction

Temperature is a critical meteorological parameter for crop development and function both in the short and long term^1^. High or low temperature, even for a short period, affects crop growth, especially in temperature-sensitive crops like wheat. Very importantly, plants’ response to changes in day and night temperature (Thermoperiodism) affects the production of various enzymes and plant growth chemicals^2^. Further, an increase in temperature increases the water vapour in the air exponentially at saturation, raising the vapour pressure deficit (VPD) causing an enhancement in water loss from plants, causing a substantial reduction in vegetation productivity and yield^3^. Therefore, shifts in long-term mean annual temperature and extreme temperature events will likely have significant impacts on crop production.

Biological development rates are linearly related to temperature. It is important to include the variations in temperature in agricultural models which describe the crop phenology and development based on heat accumulation^4^. Although daily mean temperature determines the growth and development of crop plants, the day and night cycle/diurnal pattern of temperature is more important as it affects many plant morphological characteristics like leaf, shoot orientation, plant height and internodes length etc.^5^ and disrupts the nutrient balance^6^. Therefore, analysing the response of crop plants to hourly temperature is more crucial than daily data.

Many studies revealed the need for sub-daily/hourly maximum and minimum temperatures to monitor the crop phenological events^7,8^, dormancy breaks of temperate fruits^9,10^, chilling hour requirement for apple^11–14^, forecasting of plant disease transmission^15^ and prediction of frost occurrence^16^.

Generally, meteorological observatories record daily maximum and minimum temperatures and hourly temperature data is available at a few places^17^. The period for missing observational data can be replaced with data generated by weather generators^18^. At locations, where Automatic Weather Stations (AWS) are installed even record erroneous and uneven hourly/sub-hourly weather data due to malfunctioning of sensors/unknown disruptions^19^. Moreover, the higher cost of installation and periodic maintenance of AWS makes it difficult to increase its network, especially in developing/third-world countries.

As there is a paucity of hourly temperature data, often daily maximum and minimum temperature data are used to estimate the diurnal temperature curves^20^. The diurnal temperature curves have been in a variety of ways that vary from simple curve-fitting models based upon sine curves^21–27^ to more sophisticated techniques utilizing Fourier analysis^28^ and complex energy balance models^29–31^. Even though modelling approaches are different^32–35^, most models are based on temperature. Employing statistical tools to enhance the performance of these predictive models/equations is a general practice in the research arena. Prior work with regard to the estimation of hourly temperature values based on the equations in widely used crop simulation models gave unsatisfactory results. This led to the use of bias correction technique to improve the model prediction ability in the present study.

With this background, the present study explores the accuracy of different models for estimating hourly temperatures from daily maximum and minimum temperatures at places representing various climatic and soil types. Three bias correction methods to improve the accuracy and usability of the estimated hourly temperature data was also taken up in this study.

## Results and discussion

The estimated hourly temperature from daily maximum and minimum temperature data using four different models (WAVE, PL, Soygro and Temperature) indicated appreciable error between estimated and observed data. To increase the accuracy of estimated hourly temperature data three bias correction methods (Linear Regression, Linear Scaling and Quantile Mapping) were used and the results are furnished below.

### Evaluation of bias correction methods

**Figure 1:**
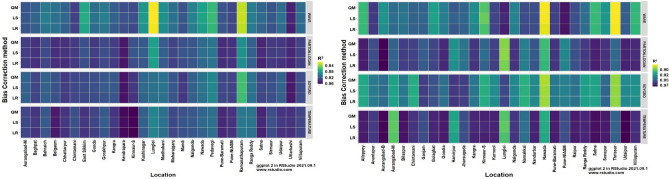
Performance of bias-corrected models *w.r.t* R^2^ across the study locations during *kharif* (left) and *rabi* (right).

**Figure 2:**
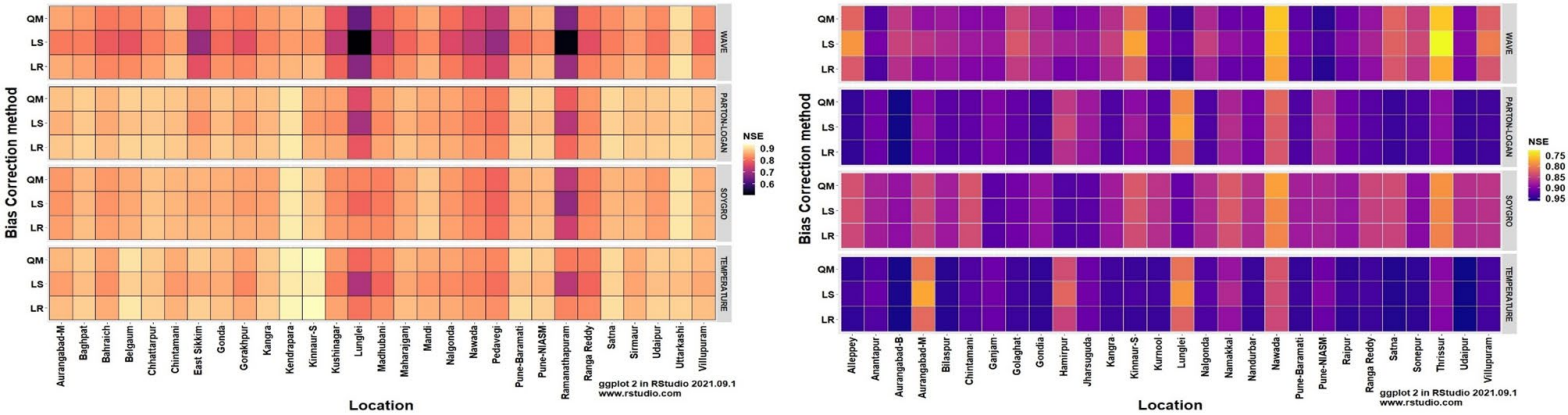
Performance of bias-corrected models *w.r.t.* NSE across the study locations during *kharif* (left) and *rabi* (right).

**Figure 3:**
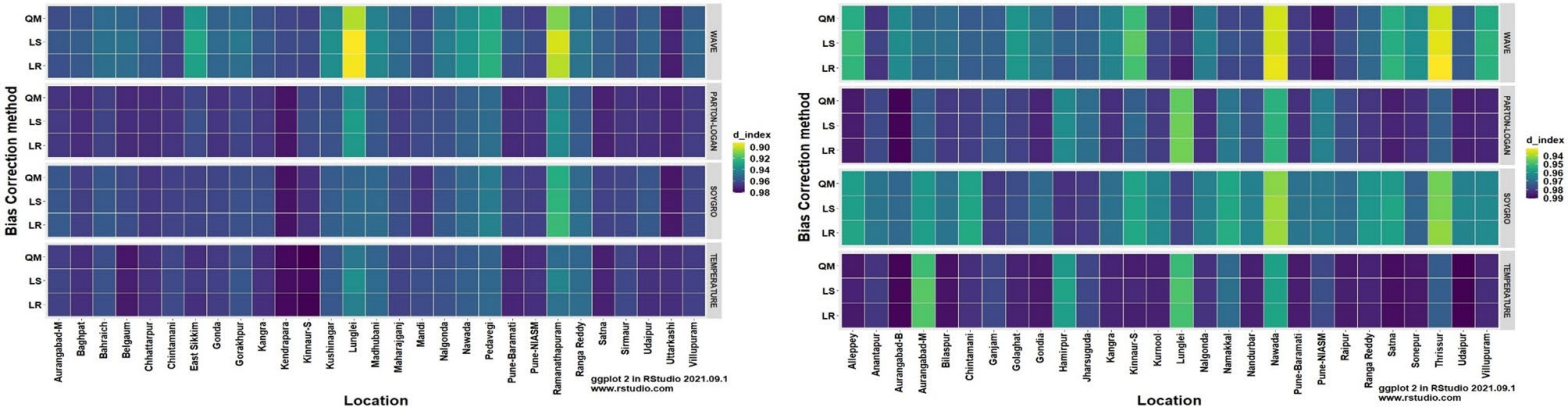
Performance of bias-corrected models *w.r.t* D-index across the study locations during *kharif* (left) and *rabi* (right).

**Figure 4:**
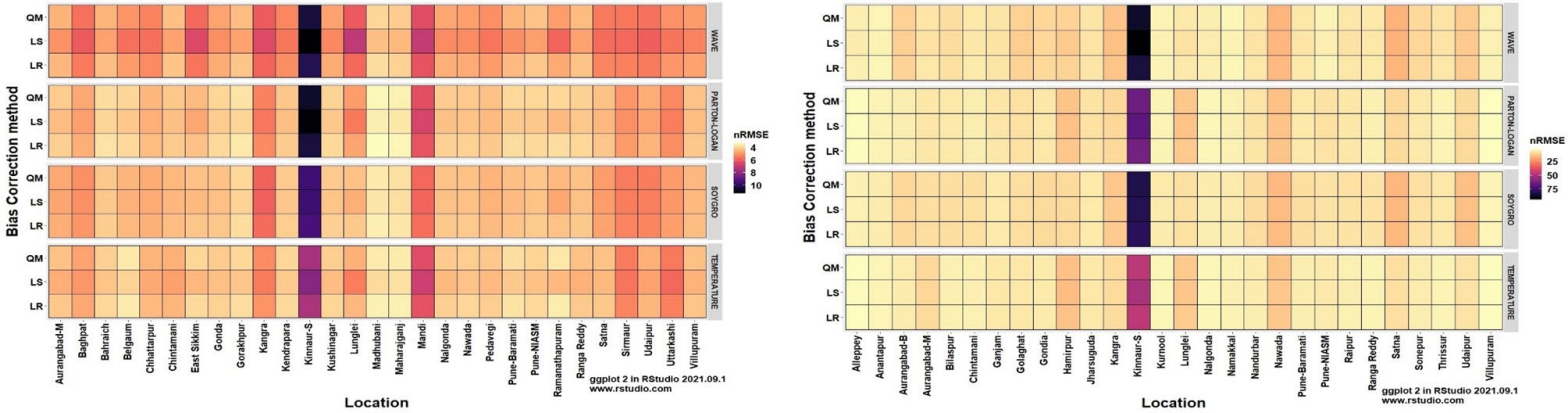
Performance of bias-corrected models *w.r.t* nRMSE across the study locations during *kharif* (left) and *rabi* (right).

**Figure 5:**
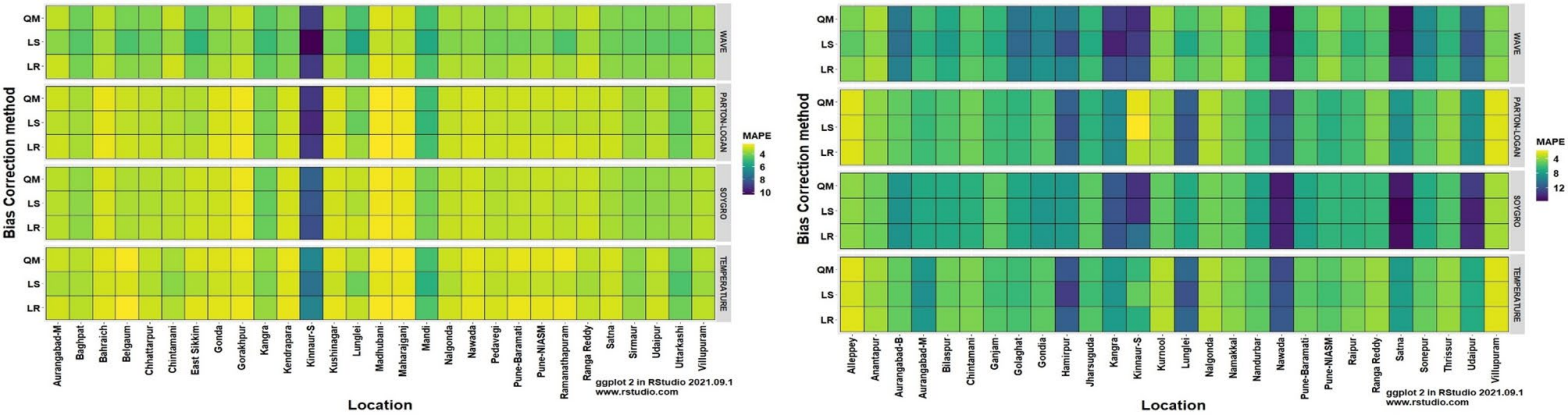
Performance of bias-corrected models *w.r.t* MAPE across the study locations during *kharif* (left) and *rabi* (right).

The heatmaps of performance metrics in terms of three efficiency criteria and two different measures for each of the 12 bias-corrected models at each location during *kharif* (2012 and 2013) and *rabi* (2012–13 and 2013–14) are presented in Figs. 1, 2, 3, 4 and 5. During the *kharif* season, the bias-corrected Soygro model exhibits higher R^2^, NSE and D-index values at 14 locations, followed by the WAVE model and Temperature models at 8 and 6 locations, respectively. The results of Reicosky et al.^36^ indicated that the Soygro model was equally robust as the WAVE model in estimating hourly temperature from daily maximum and minimum temperature in most circumstances. Across these 28 locations, bias correction using Linear Regression was found to be more appropriate in estimating the hourly temperature. In the case of difference measures (nRMSE), Soygro model with Linear Regression as biascorrection appears to be most accurate in estimating the hourly temperature at 14 locations, followed by the WAVE and Temperature models at 8 and 6 locations, respectively, which is the same as that of the index measures. MAPE value for bias corrected (Linear Regression) Temperature model was less at 12 locations, followed by the Soygro and WAVE models at 9 and 7 locations, respectively. Table 1 shows the best of the bias-corrected models in terms of efficiency criteria and difference measures across each of the study locations.

**Table 1:** Best of bias-corrected models for *kharif* season in terms of efficiency criteria and difference measures across each of the study locations.

Locations	Efficiency criteria			Difference measures		Mode
	R^2^	NSE	D-Index	nRMSE	MAPE	
Bahraich, Gonda, Gorakhpur, Kushinagar Madhubani, Pedavegi, Ramanathapuram and Ranga Reddy	S-LR	S-LR	S-LR	S-LR	S-LR	S-LR
Baghpat, Chhattarpur, Kendrapara, Nawada, Udaipur and Villupuram	S-LR	S-LR	S-LR	S-LR	T-LR	S-LR
East Sikkim, Kangra, Maharajganj, Nalgonda and Satna	T-LR	T-LR	T-LR	T-LR	T-LR	T-LR
Kinnaur	T-LR	T-LR	T-LR	T-LR	S-LR	T-LR
Aurangabad-M, Belgaum, Chintamani, Kinnaur, Lunglei, Mandi, Pune and Uttarkashi	W-LR	W-LR	W-LR	W-LR	W-LR	W-LR
Sirmaur	W-LR	W-LR	W-LR	W-LR	T-LR	W-LR

S, Soygro; T, Temperature; W, Wave; LR, Linear Regression.

Variation among the models in different methods of error analysis was not consistent, especially with respect to MAPE. Therefore, a greater number of efficiency criteria and different measures are chosen for error analysis to find out an appropriate bias-corrected model for estimating the hourly temperature at any particular location.

During the *rabi* season, the Temperature model with LR bias-correction was found to be accurate at more locations (20), followed by WAVE and Soygro models at 5 and 2 locations, respectively with respect to both indices (R^2^, D-index and NSE) and difference measures (nRMSE and MAPE). The study of Cesaraccio et al.^7^ also revealed that the estimated hourly temperature data by Temperature model was superior over PL, WAVE and Wilkerson model^37^. Baker et al.^38^ also reported that all three methods (WAVE, PL and Linear model) performed equally well in estimating hourly temperature data from daily maximum and minimum temperature data. However, the above paper reported that all three methods performed well during summer when compared to winter. The best of the bias-corrected models in terms of efficiency criteria and different measures across the study locations during *rabi* season is shown in Table 2. It is to be noted that, LR was found to be more effective in reducing the bias-correction of the appropriate model at each location. We used the LR criterion with an assumption that there are no measurement errors in the observed data, as hourly data was collected from AWS.

**Table 2:** Best of the bias-corrected models for *rabi* season terms of efficiency criteria and difference measures across each of the study locations.

Locations	Efficiency criteria			Difference measures		Mode
	R^2^	NSE	D-index	nRMSE	MAPE	
Hamirpur	S-LR	S-LR	S-LR	S-LR	S-LR	S-LR
Nawada	S-LR	S-LR	S-LR	S-LR	T-LR	S-LR
Anantapur, Aurangabad-M, Lunglei and Namakkal	W-LR	W-LR	W-LR	W-LR	W-LR	W-LR
Alleppey, Aurangabad-B, Bilaspur, Chintamani, Golaghat, Gondia, Kangra, Kurnool, Nalgonda, Nandurbar, Pune-Baramati, Ranga Reddy, Raipur, Satna, Sonepur, Thrissur, Udaipur and Villupuram	T-LR	T-LR	T-LR	T-LR	T-LR	T-LR
Ganjam and Jharsuguda	T-LR	T-LR	T-LR	T-LR	S-LR	T-LR
Kinnaur	T-LR	T-LR	T-LR	T-LR	PL-LS	T-LR

S, Soygro; T, Temperature; W, Wave; LR, Linear Regression.

### Comparison of mean hourly temperature

**Figure 6:**
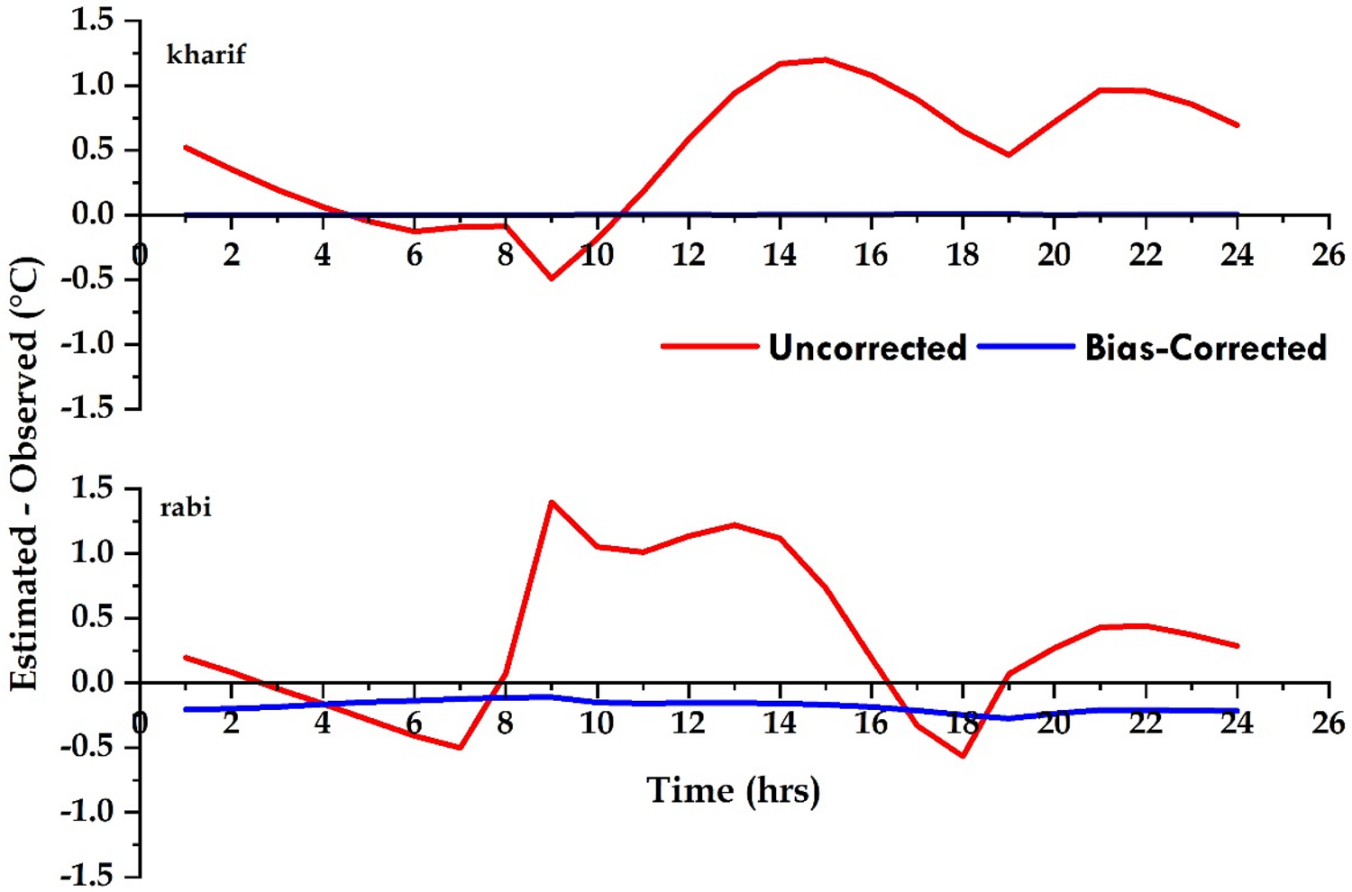
Average hourly temperature error calculated by comparing estimated and observed hourly temperature during *kharif* and *rabi* seasons.

To compare the appropriate method, with the observed data, before and after bias correction a line plot of mean hourly temperature for both *kharif* and *rabi* seasons was plotted and presented in Fig. S1 and Fig. S2 (Supplementary file), respectively at each location. The estimated hourly temperature with and without bias correction shows a clear difference in estimating the hourly temperature, during both *kharif* and *rabi* seasons. The LR bias-correction method explicitly shows the improvement in estimating the hourly temperature across each location. The estimated hourly temperature, after bias correction, is reasonably close to the observed values during both *kharif* and *rabi* seasons (Figs. 1, 2, 3, 4, 5). McDonnel et al.^39^ found LR method to be the most appropriate bias correction method for both air and soil temperatures, which improved the air and soil temperature forecasts given by European Centre for Medium-Range Weather Forecasts for Ireland. The magnitude of the errors with both uncorrected and bias-corrected models seems to vary throughout the 24 h at each location and is more distinctly noted by plotting the average hourly error, *i.e.* the difference between the estimated temperature, with and without bias-correction, and the observed temperatures in Fig. 6. The pooled data analysis showed that, during the *kharif* season, the difference between estimated (uncorrected and bias-corrected) and observed error is smallest during 04 to 08 h as most of the models take Tmin as input, whereas during the *rabi* season it is observed from 03 to 08 h in the morning. Errors with the uncorrected models at other times of the day are as large as − 1.2 °C during *kharif*, and − 1.4 °C during *rabi*. Reicosky et al.^36^ also observed the least variation between estimated and observed hourly temperature when minimum temperature was recorded and the highest was observed during other times of the day. Further, they reported that maximum temperature didn’t appear to affect the accuracy of the models employed to estimate hourly temperature from daily data. After bias correction, the magnitude of error (difference in model output and observed) got reduced to ~ 0 °C in *kharif*, whereas still underestimated by 0.2 °C in *rabi.* This could be attributed to the low diurnal variation in temperature that occurs during *kharif* season due to cloudy/overcast conditions in most of the day. Whereas, *rabi* is the driest season of the year during which mostly clear days and nights are observed. Reicosky et al.^36^ also found that predicted hourly temperature data from daily maximum and minimum temperatures fitted well during cloud-free days than cloudy days.

### Suitability of models in various climatic and soil types during *kharif* and *rabi* seasons

#### Climate type

During *kharif* season, Soygro model was found to be performing well in estimating the hourly temperature in hot sub-humid (7 locations) and hot semi-arid (7 locations) climates. However, in locations representing warm per-humid/sub-humid to hot semi-arid climates WAVE and Temperature models performed better (Table 3). These observations indicate a mixed performance of the models in different climates. *Kharif* is the main rainy season of the year and prevailing cloudy conditions/overcast sky during the season leads to low diurnal fluctuation in temperature^40^. An overcast sky disturb/interfere with the incoming radiant energy during day hours and especially energy loss during night hours in the form of longwave radiation. This leads to a change in the diurnal pattern of air temperature i.e. reduction in the difference between daytime and nighttime. It is understood from the detail of the respective models that Soygro and Temperature models have divided 24 h of a day into three parts while WAVE and PL models divided the day into two parts (details in Materials and Methods section). Soygro model could capture well the change in the diurnal pattern of temperature, as two out of the three equations in the model consider data during midnight to sunrise hours and sunset to midnight hours. This could be the reason for the better performance of Soygro model in many locations when compared to WAVE and Temperature models.

**Table 3:** Performance of models in predicting hourly temperature in different climates during *kharif* and *rabi* seasons.

Climate type	*Kharif* season (28 locations)				*Rabi* season (27 locations)			
	WAVE model	PL model	Soygro model	Temperature model	WAVE model	PL model	Soygro model	Temperature model
Warm Per-humid	1	–	–	–	1	–	–	–
Hot humid and sub-humid	–	–	–	1	–	–	–	2
Warm sub-humid	3	–	–	2	–	–	1	2
Hot sub-humid	–	–	7	2	–	–	1	9
Hot semi-arid	4	–	7	1	2	–	–	8
Hot arid	–	–	–	–	1	–	–	–

During *rabi* season, the Temperature model exhibited better performance in 21 out of 27 locations falling under different AERs from hot per-humid & humid to hot semi-arid climates. It is observed that Soygro model worked well only in two locations representing hot and warm sub-humid climates. WAVE model was found to be best-suited for one per-humid, one arid and two hot semi-arid locations. *Rabi* season is the driest season of the year characterised by low atmospheric humidity, cloud-free sky and a higher diurnal temperature range^40^. The Temperature model, comprising two equations dealing with daylight hours and one equation for night hours, probably captured the hourly march of temperature well during the *rabi* season. It was also observed that the PL model could not perform better either during *kharif* or *rabi* seasons in any of the locations. One out of two equations of the PL model, which deals with night hours, assumes temperature decrement from sunset to sunrise at an exponential rate. Probably, this assumption can be attributed to the poor performance of PL model across locations.

#### Soil type

Soil colour and texture play a predominant role in governing the diurnal pattern of soil temperature, which in turn affects the atmospheric temperature through latent and sensible heat fluxes^41^. The performance of models in estimating the hourly temperature for locations having different soil types was looked at. The results indicated that Soygro model performed better in locations having alluvium-derived soils, especially during *kharif* season. However, Temperature and WAVE models did not show any specific pattern for soil types. During *rabi* season, the hourly air temperature was well predicted by the Temperature model in all soil types. The Temperature model worked well in 11 out of 27 locations represented by red, black, and lateritic soils (Table 4). Performance of the model was also good at 6 locations having alluvium soils and 2 locations each represented by red loamy and brown forest podzolic soils. At the same time, the WAVE model performed well in red, black, lateritic and red loamy soils, while Soygro model performed well in only two locations represented by alluvium and brown forest soils. The study showed that Soygro and Temperature models worked well in most of the locations during *kharif* and *rabi* seasons, respectively when compared to the WAVE model. However, the PL model’s performance was not up to the mark, compared to the other three models in any of the soil types. It is understood from the results that Soygro and Temperature models which have three different equations to deal with the day and night hours helped for precise estimation of hourly data by these models.

**Table 4:** Performance of models in predicting hourly temperature in different soil types during *kharif* and *rabi* seasons.

Soil type	*Kharif* season (28 locations)				*Rabi* season (27 locations)			
	WAVE model	PL model	Soygro model	Temperature model	WAVE model	PL model	Soygro model	Temperature model
Alluvium derived	–	–	11	2	–	–	1	6
Red, black and lateritic	4	–	2	2	3	–	–	11
Red loamy	1	–	1	–	1	–	–	2
Brown forest podzolic	3	–	–	2	–	–	1	2

As mentioned earlier, the Soygro model performed well in the locations with alluvium soils, especially during *kharif* season and not during the *rabi* season. In general, the average soil moisture status of the soil is higher during the *kharif* season than the *rabi* season and vice versa. During the *kharif* season, when soil moisture nears field capacity, it takes more time to heat up the soil and also the atmosphere later as the specific heat of wet sandy soil is almost two times higher (0.4 cal g^−1^ deg^−1^) than dry sandy soil (0.2 cal g^−1^ deg^−1^)^42^. During the *rabi* season, as soil is dry, it absorbs and releases the heat energy in less time and also the diurnal soil temperature range would be greater than the *kharif* season. Though Soygro and Temperature models use three equations, Soygro model consists of only one equation to deal with the daylight period while the Temperature model has two equations for the daylight period. This could be the reason for the better performance of the Temperature model during *rabi* season in most of the locations having different soil types.

## Conclusions

Four models viz*,* WAVE, PL, Soygro and Temperature model were used in estimating the hourly temperature at selected locations in different AERs of the country during two crop seasons (*kharif* and *rabi*). When compared with the hourly observed data, the bias-corrected Soygro model exhibits higher values *w.r.t* three efficiency criteria (R^2^, NSE and D-index) at 14 locations, followed by the WAVE model and Temperature models in 8 and 6 locations, respectively during the *kharif* season. In the case of the *rabi* season, bias corrected Temperature model appears to be accurate at more locations (20), followed by WAVE and Soygro models at 5 and 2 locations, respectively. Bias correction was found to be more effective in reducing the error of the appropriate model at each location with Linear Regression irrespective of the season. The magnitude of errors with both uncorrected and bias-corrected models seems to be changing over the 24-h period of the day across each of the locations. The performance of four different models over different agroecological regions of the country revealed the better performance of the Soygro model during the *kharif* season at locations representing hot sub-humid and semi-arid climates. Whereas, the Temperature model outperformed the other model during the *rabi* season at most of the locations ranging from hot per-humid to hot semi-arid climates. Estimating the accuracy of hourly temperature in different soil types indicated that the Soygro model worked well during the *kharif* season at the locations having alluvium-derived soils. At the same time, during the *rabi* season, the performance of the Temperature model was better than other models in the agro ecological regions dominated by red, black and lateritic soils. The smallest difference between estimated (uncorrected and bias-corrected) and observed was noticed from 04 to 08 h during the *kharif* season when most of the models assume Tmin as input, while it was from 03 to 08 h in the morning during the *rabi* season. The results of the study strongly suggested that hourly temperature during the *kharif* and *rabi* seasons can be estimated using the Soygro and Temperature models, respectively after the bias correction by the Linear Regression method, as these two models estimated hourly temperature with better accuracy at a majority of the locations representing different climate and soil types. Nevertheless, the WAVE model also can be used to estimate the hourly temperature data at some of the study locations. However, the PL model did not perform satisfactorily at any of the locations. Hourly temperature data in lieu of daily temperature (maximum and minimum) data would enhance the precision in predicting phenological and other biological events.

## Data and methods

### Study area

**Figure 7:**
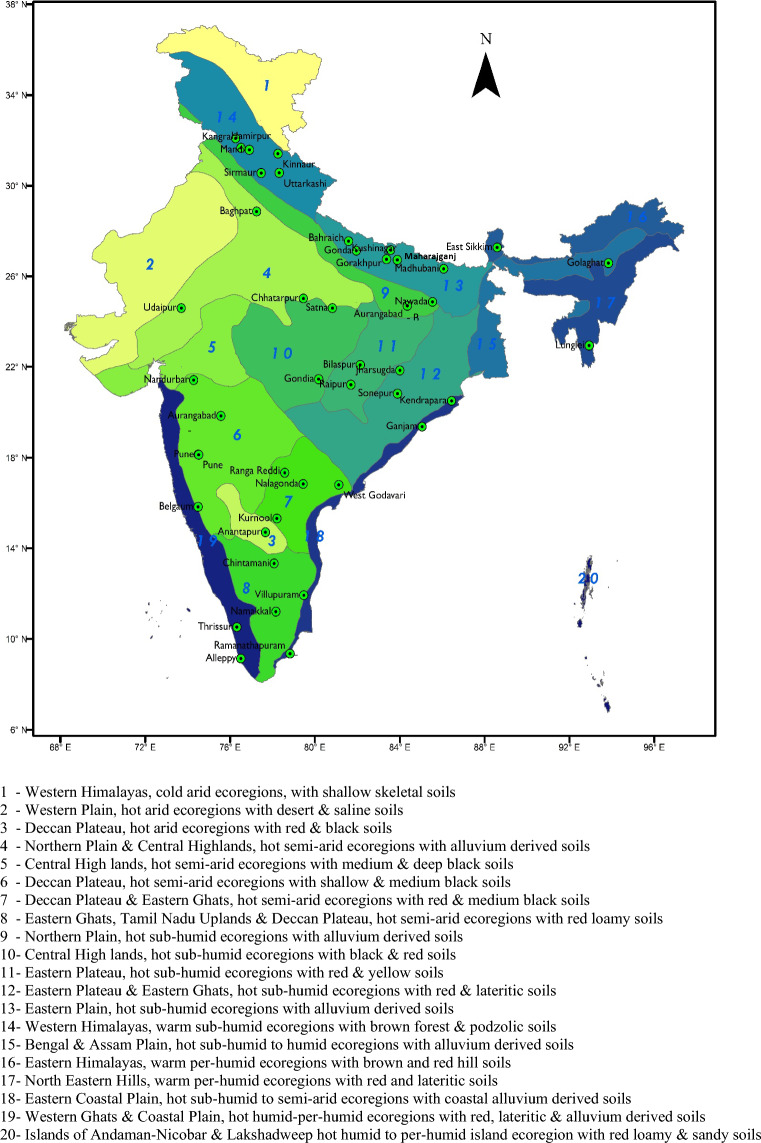
Study locations over different AER’s of the country *(Numbers within each polygon represent different AERs)* (map generated using ArcMap 10.3; URL: https://www.esri.com/en-us/arcgis/products/arcgis-desktop/overview).

Observed maximum and minimum temperatures, at daily and hourly scales, were collected from the AWS of the AICRPAM-NICRA network. National Innovations in Climate Resilient Agriculture (NICRA), a network project of the Indian Council of Agricultural Research (ICAR) aims to enhance the resilience of Indian agriculture to climate change and climate vulnerability through strategic research and technology demonstration in the 100 most vulnerable districts of the country. The climatic variability is being assessed by the All India Coordinated Research Project on Agrometeorology (AICRPAM) by installing AWS in each district under the NICRA project^43^. The AWS records 6 meteorological parameters viz*.,* temperature (maximum and minimum), relative humidity (maximum and minimum), wind speed, wind direction, rainfall and solar direction. Depending on the availability of continuous hourly temperature (maximum and minimum) data over the study period, 44 locations were selected for the present study (Fig. 7 and Table S1 of the Supplementary file). Out of 44 locations, the hourly temperature was estimated at 28 locations during the *kharif* season (2012 and 2013) and at 27 locations during the *rabi* season (2012–13 and 2013–14).

### Methods of estimating hourly temperature

Four different methods for estimating hourly temperature were tested at different agro-ecological regions (AERs) of the country during two major crop seasons viz*.,* the *kharif* (15-Jun to 15-Oct) and *rabi* (15-Oct to 15-Mar), by inputting the daily maximum and minimum temperature. These methods were taken from different crop growth simulation models. Therefore, in the present study, these methods are known by the name of their respective crop growth models or the sub-routines in which they were employed. The details of each method are discussed hereunder.

### WAVE model

The model was first introduced by De Wit et al.^44^ and it was included in the subroutine WAVE in ROOTSIMU V4.0^45^. The model uses two different relations; formula one for estimating the hourly temperature from the time of minimum temperature (sunrise hour) to the time of maximum temperature (1400 h) and another for estimating from the time of maximum temperature to the time of the minimum temperature of the next day. A cosine function is used in the model for both periods and is given by the following Eqs. (1) and (2).

For Sunrise Hour ≤ H ≤ 1400 h

1 $$ {\text{T}}_{{({\text{H}})}} = {\text{ T}}_{{{\text{ave}}}} {-}{\text{ T}}_{{{\text{amp}}}} *({\text{Cos }}[\pi*\left( {{\text{H-Sunrise Hour}}} \right)/ \, \left( {{\text{14-Sunrise Hour}}} \right)]) $$

For 1400 h < H < Sunrise Hour of next day

2 $$ {\text{T}}_{{({\text{H}})}} = {\text{ T}}_{{{\text{ave}}}} + {\text{ T}}_{{{\text{amp}}}} *({\text{Cos }}[\pi*\left( {{\text{H}}^{\prime } } \right)/\left( {{1}0 + {\text{ Sunrise Hour}}} \right)]) $$

where T_(H)_ is the temperature at hour H, ${\mathrm{T}}_{\mathrm{ave}}=\frac{{\mathrm{T}}_{\mathrm{max}}+{\mathrm{T}}_{\mathrm{min}}}{2}$ ; ${\mathrm{T}}_{\mathrm{amp}}=\frac{{\mathrm{T}}_{\mathrm{max}}-{\mathrm{T}}_{\mathrm{min}}}{2}$; T_max_ and T_min_ are the maximum and minimum temperature, respectively. H’ = H-14 if H > 1400 h and H’ = H + 10 if H < Sunrise Hour. For estimating the hourly temperature past 1400 h, T_min_ of the next day is considered. In the WAVE model, no site-specific calibration is required.

### Parton and Logan (PL) model

The model developed by Parton and Logan^46^ uses two different equations, like in the WAVE model, for estimating the hourly air temperature during daylight hours and night-time hours. Parton-Logan’s model considers the sunrise hour to sunset hour as daylight hours and the time between the sunset hour and sunrise hour of the next day as night-time hours. The model utilizes a truncated sine wave at daylight hours (day-time) and an exponential decrease in temperature at night-time for estimating the hourly temperature. The temperature at any given hour (T_(H)_) during the daylight hours is given by Eq. (3).

3 $$ {\text{T}}_{{({\text{H}})}} = {\text{ T}}_{{{\text{min}}}} + \, \left( {{\text{T}}_{{{\text{max}}}} - {\text{ T}}_{{{\text{min}}}} } \right) \, *{\text{ Sin }}\left[ {\frac{{\uppi }}{2}{*}\frac{{\left( {{\text{H}} - {\text{H}}_{{{\text{min}}}} } \right)}}{{\left( {{\text{H}}_{{{\text{max}}}} - {\text{H}}_{{{\text{min}}}} } \right)}}} \right] $$

where T_max_ and T_min_ are the maximum and minimum temperature, respectively and H_max_ and H_min_ are the hour of maximum and minimum temperatures, respectively. In the present study, H_max_ is considered as 4 h before sunset hour (i.e. H_max_ = H_ss_-4 where H_ss_ is the sunset hour) and sunrise hour is considered as the H_min_. The temperature at any given hour (T_(H)_) during the night-time hours is given by Eq. (4).

4 $$ {\text{T}}_{{({\text{H}})}} = {\text{ T}}_{{{\text{min}}}} + \, \left( {{\text{T}}_{{{\text{ss}}}} - {\text{ T}}_{{{\text{min}}}} } \right) \, *e^{{ - {\text{b*}}\frac{{\text{N}}}{{\text{L}}}}} $$

where T_ss_ is sunset hour temperature which can be determined using the daytime hour temperature relation, b is the empirical constant (dimensionless), N is the time since sunset (s) and L is the night length (s). In the present study, the empirical constant ‘b’ is taken as 2.20 across all the locations, as the parameter did not appear to be strongly site-specific^36^.

### Soygro model

The model introduced by Wilkerson et al.^37^ estimates the hourly temperature by dividing the day into three segments viz*.,* midnight to sunrise + 2 h; daylight hours and sunset to midnight. For each segment, the model uses three different Eqs. (5) to (14), which are given below.

For midnight to sunrise + 2 h

5 $$ {\text{TAU }} = \left( {{\text{SET}}_{{{\text{n}} - {1}}} {-}{\text{ RISE}}_{{{\text{n}} - {1}}} - { 2}} \right)/ \, \left( {{\text{SET}}_{{{\text{n}} - {1}}} {-}{\text{ RISE}}_{{{\text{n}} - {1}}} } \right) $$

6 $$ {\text{TLIN }} = {\text{ TMIN}}_{{{\text{n}} - {1}}} + \, \left( {{\text{TMAX}}_{{{\text{n}} - {1}}} {-}{\text{ TMIN}}_{{{\text{n}} - {1}}} } \right) \, *{\text{ Sin }}\left( {{\text{TAU}}} \right) $$

7 $$ {\text{SLOPE }} = \, \left( {{\text{TLIN }} - {\text{ TMIN}}_{{\text{n}}} } \right) \, / \, \left( {{24} - {\text{SET}}_{{{\text{n}} - {1}}} + {\text{ RISE}}_{{\text{n}}} + { 2}} \right) $$

8 $$ {\text{T}}_{{({\text{H}})}} = {\text{ TLIN }}{-}{\text{ SLOPE }}\left( {{\text{H }} + { 24 }{-}{\text{ SET}}_{{{\text{n}} - {1}}} } \right) $$

For sunset to midnight

9 $$ {\text{TAU }} = \left( {{\text{SET}}_{{\text{n}}} {-}{\text{ RISE}}_{{\text{n}}} - { 2}} \right)/ \, \left( {{\text{SET}}_{{\text{n}}} {-}{\text{ RISE}}_{{\text{n}}} } \right) $$

10 $$ {\text{TLIN }} = {\text{ TMIN}}_{{\text{n}}} + \, \left( {{\text{TMAX}}_{{\text{n}}} {-}{\text{ TMIN}}_{{\text{n}}} } \right) \, *{\text{ Sin }}\left( {{\text{TAU}}} \right) $$

11 $$ {\text{SLOPE }} = \, \left( {{\text{TLIN }} - {\text{ TMIN}}_{{{\text{n}} + {1}}} } \right) \, / \, \left( {{24} - {\text{SET}}_{{\text{n}}} + {\text{ RISE}}_{{{\text{n}} + {1}}} + { 2}} \right) $$

12 $$ {\text{T}}_{{({\text{H}})}} = {\text{ TLIN }}{-}{\text{ SLOPE }}\left( {{\text{H }}{-}{\text{ SET}}_{{\text{n}}} } \right) $$

For daylight hours

13 $$ {\text{TAU }} = \left( {{\text{H }}{-}{\text{ RISE}}_{{\text{n}}} - { 2}} \right)/ \, \left( {{\text{SET}}_{{\text{n}}} {-}{\text{ RISE}}_{{\text{n}}} } \right) $$

14 $$ {\text{T}}_{{({\text{H}})}} = {\text{ TMIN}}_{{\text{n}}} + \, \left( {{\text{TMAX}}_{{\text{n}}} {-}{\text{ TMIN}}_{{\text{n}}} } \right) \, *{\text{ Sin }}\left( {{\text{TAU}}} \right) $$

where TAU, TLIN, SLOPE are temporary variables in calculations, T_(H)_ is the temperature at hour H, n is the current day of the year (1 to 365), RISE is the sunrise hour and SET is the sunset hour. The method assumes a change from night to day temperature at sunrise + 2 h, and the night temperatures are linear with time. It is to be noted that, like the WAVE and Parton-Logan models, the Soygro model also does not consider any site-specific constants.

### Temperature model

The temperature model presented by Cesaraccio et al.^7^ is an empirical model for estimating the hourly mean temperature. Like the Soygro model, the temperature model also divides the day into three time periods i.e. from the sunrise hour (H_n_) to the time of maximum temperature (H_x_), from H_x_ to the sunset hour (H_o_) and from H_o_ to the sunrise hour for the next day (H_p_). The model uses two sine-wave functions in the daylight and a square-root decrease in temperature at night. The sunrise and sunset hours are determined as a function of site latitude and the day of the year, whereas the hour of maximum temperature is assumed to be 4 h before sunset (H_x_ = H_o_-4). H_p_ is calculated as H_p_ = H_n_ + 24. T_n_ and T_x_ are the minimum and maximum temperature of the current day which occurs at H_n_ and H_x_, respectively while the temperature during the sunset hour (T_o_) is determined using the below-mentioned Eq. (15).

15 $$ {\text{T}}_{{\text{o}}} = {\text{ T}}_{{\text{x}}} {-}{\text{ c }}\left( {{\text{T}}_{{\text{x}}} - {\text{ T}}_{{\text{p}}} } \right) $$

where T_p_ is the minimum temperature of the following day and c is the empirical constant, which is obtained by fitting the equation to the observed hourly data set. For a given T_n_, T_x_, T_o_ and T_p_, the Temperature model estimates the temperature at any given hour (T_(H)_) for the three-time periods with the help of respect to three different Eqs. (16) to (18).

For H_n_ < H ≤ H_x_

16 $$ {\text{T}}_{{({\text{H}})}} = {\text{T}}_{{\text{n}}} + \alpha *{\text{Sin}}\left[ {\left( {\frac{{{\text{H}} - {\text{H}}_{{\text{n}}} }}{{{\text{H}}_{{\text{x}}} - {\text{H}}_{{\text{n}}} }}} \right)\frac{\pi }{2}} \right] $$

For H_x_ < H < H_o_

17 $$ {\text{T}}_{{({\text{H}})}} = {\text{ T}}_{{\text{o}}} + {\text{ R}}*{\text{Sin}}\left[ {\frac{\pi }{2} + \left( {\frac{{{\text{H}} - {\text{H}}_{{\text{x}}} }}{4}} \right)\frac{\pi }{2}} \right] $$

For H_o_ < H ≤ H_p_

18 $$ {\text{T}}_{{({\text{H}})}} = {\text{T}}_{{\text{o}}} + {\text{ b}}\sqrt {{\text{H}} - {\text{H}}_{{\text{o}}} } $$

where $$\alpha $$ = T_x_ – T_n_; R = T_x_ – T_o_ and b = $$\frac{{\mathrm{T}}_{\mathrm{p}}-{\mathrm{T}}_{\mathrm{o}}}{\sqrt{{\mathrm{H}}_{\mathrm{p}}-{\mathrm{H}}_{\mathrm{o}}}}$$.

### Bias correction methods

Researchers used different bias correction methods to reduce the errors and improve the accuracy of prediction significantly in different parts of the world^47–50^. In the present study also, to reduce the biases and improve the accuracy of estimated hourly temperature, three bias correction methods were used and the details of each are discussed below.

### Linear regression

Linear Regression (LR) is simply a line of best fit through the standard regression plot of the observed against the estimated. Since temperature is well described by a normal distribution, a linear fit is sufficient. The relative relationship of the values being compared is given by the slope value, whereas the intercept indicates the lead or lag between the data. A slope of one and a y-intercept of zero show a perfect fit between simulated and observed data^51^ as mentioned in Eq. (19).

19 $$ {\text{T}}_{{{\text{cor}},{\text{m}},{\text{d}},{\text{h}}}} = {\text{ m}}_{{{\text{m}}({\text{h}})}} *{\text{ T}}_{{{\text{est}},{\text{m}},{\text{d}},{\text{h}}}} + {\text{ c}}_{{{\text{m}}({\text{h}})}} $$

where T_cor,m,d,h_ and T_est,m,d,h_ are the bias-corrected and estimated temperature (using either of the four models), respectively for the ‘h^th^’ hour of ‘d^th^’ day and ‘m^th^’ month, m_m(h)_ and c_m(h)_ are the slope and intercepts of ‘h^th^’ hour of ‘m^th^’ month, respectively.

### Linear scaling

The Linear Scaling (LS) method aims to perfectly match the monthly mean of corrected values with that of observed values^52^. It operates with monthly correction values worked out as the difference between observed and simulated data. In the present study, the bias correction for the estimated hourly temperature is given by Eq. (20).

20 $$ {\text{T}}_{{{\text{cor}},{\text{m}},{\text{d}},{\text{h}}}} = {\text{T}}_{{{\text{est}},{\text{m}},{\text{d}},{\text{h}}}} + \mu \, \left( {{\text{T}}_{{{\text{obs}},({\text{m}}({\text{h}})}} } \right) - \mu \, \left( {{\text{T}}_{{{\text{est}},{\text{m}}({\text{h}})}} } \right) $$

where μ (T_obs,m(h)_) and μ (T_est,m(h)_) are the mean value of observed and estimated temperature for the ‘h^th^’ hour of ‘m^th^’ month.

### Quantile mapping

Among the different bias correction methods, Quantile Mapping (QM) is considered the most useful and popular. By applying a transfer function, QM corrects the systematic bias in the simulated data so that it matches the distribution of the observational dataset^53,54^. For the estimated hourly temperature, the QM bias correction is given by Eq. (21).

21 $$ {\text{T}}_{{{\text{cor}},{\text{m}},{\text{d}},{\text{h}}}} = {\text{CDF}}_{{{\text{obs}},{\text{m }}}}^{ - 1} \left( {{\text{CDF}}_{{{\text{est}},{\text{m}}}} \left( {{\text{T}}_{{{\text{est}},{\text{m}},{\text{d}},{\text{h}}}} } \right)} \right) $$

where T_cor,m,d,h_ and T_est,m,d,h_ are the bias-corrected and estimated temperature (using either of the four models), respectively for the ‘h^th^’ hour of ‘d^th^’ day and ‘m^th^’ month; CDF^−1^_obs,m_ is inverse CDF of observed data set in the validation period; CDF_est,m_ is CDF of estimated data set in the validation period.

### Methods of error analysis

After applying bias correction techniques (LR, LS and QM), the accuracy of various models (WAVE, Parton-Logan, Soygro and Temperature) in estimating the hourly temperature was tested by comparing them with the observed values at each location. The “goodness of fit” of each model was assessed using three efficiency criteria (coefficient of determination, coefficient of efficiency and index of agreement) and two different measures (normalized root mean square error and mean absolute percentage error). The bias-corrected model having more efficiency criteria and fewer difference measures is chosen as the appropriate model for estimating the hourly temperature at a particular location. The details of each efficiency criterion and different measures are given below.

### Coefficient of determination

The coefficient of determination (R^2^) is the square of Pearson's product-moment correlation coefficient and describes the proportion of the total variance in the observed data that can be explained by the model. R^2^ value ranges from 0 to 1, with higher values indicating better agreement and the formula of R^2^ is given in Eq. (22).

22 $${\mathrm{R}}^{2}=\boldsymbol{ }{\left[\frac{\sum_{\mathrm{i}=1}^{\mathrm{n}}\left({\mathrm{O}}_{\mathrm{i}}-{\mathrm{O}}_{\mathrm{avg}}\right)\left({\mathrm{S}}_{\mathrm{i}}-{\mathrm{S}}_{\mathrm{avg}}\right)}{{\left[{\sum_{\mathrm{i}=1}^{\mathrm{n}}\left({\mathrm{O}}_{\mathrm{i}}-{\mathrm{O}}_{\mathrm{avg}}\right)}^{2}\right]}^{0.5}{\left[{\sum_{\mathrm{i}=1}^{\mathrm{n}}\left({\mathrm{S}}_{\mathrm{i}}-{\mathrm{S}}_{\mathrm{avg}}\right)}^{2}\right]}^{0.5}}\right]}^{2}$$

where O_i_ and S_i_ and are the observed and bias-corrected estimated values, O_avg_ and S_avg_ are the average values of observed and bias-corrected estimated values, respectively and n is the total number of observations.

### Coefficient of efficiency

As correlation-based measures are more sensitive to outliers, the coefficient of determination measure leads to a bias towards the extreme events if employed in model evaluation. Nash and Sutcliffe^55^ defined the coefficient of efficiency (NSE) as an improvement over the coefficient of determination for model evaluation purposes. Physically, it is the ratio of the mean square error to the variance in the observed data, subtracted from unity (Eq. 23).

23 $$ {\text{NSE}} = {1} - \frac{{\mathop \sum \nolimits_{{{\text{i}} = 1}}^{{\text{n}}} \left( {{\text{O}}_{{\text{i}}} - {\text{S}}_{{\text{i}}} } \right)^{2} }}{{\mathop \sum \nolimits_{{{\text{i}} = 1}}^{{\text{n}}} \left( {{\text{O}}_{{\text{i}}} - {\text{O}}_{{{\text{avg}}}} } \right)^{2} }} $$

The value of NSE ranges from − ∞ to 1, with higher values indicates perfect simulation.

### Index of agreement

In order to overcome the insensitivity of correlation-based measures to differences in the observed and model-simulated means, Willmott^56^ introduced the index of agreement (D-index), given by Eq. (24).

24 $$ {\text{D-index}} = {1} - \frac{{\mathop \sum \nolimits_{{{\text{i}} = 1}}^{{\text{n}}} \left( {{\text{O}}_{{\text{i}}} - {\text{S}}_{{\text{i}}} } \right)^{2} }}{{\mathop \sum \nolimits_{{{\text{i}} = 1}}^{{\text{n}}} \left( {\left| {{\text{S}}_{{\text{i}}} - {\text{O}}_{avg} } \right| + \left| {{\text{O}}_{{\text{i}}} - {\text{O}}_{avg} } \right|} \right)^{2} }} $$

Like R^2^, the value of D-index also varies from 0 to 1. The value of 1 indicates better agreement between model simulated and observed values.

### Normalized root mean square error

Root mean square error (RMSE) is increasingly being used in the comparison and evaluation of simulation models and considered as the paramount measure of accuracy. It is defined in the Eq. (25).

25 $$\mathrm{RMSE}=\sqrt{\sum_{\mathrm{i}=1}^{\mathrm{n}}\frac{{({\mathrm{S}}_{\mathrm{i}}-{\mathrm{O}}_{\mathrm{i}})}^{2}}{\mathrm{n}}}$$

In terms of percentage error, root mean square error is given by normalized RMSE (nRMSE), which is given by the Eq. (26).

26 $$\mathrm{nRMSE}=\left(\frac{\mathrm{RMSE}}{{\mathrm{O}}_{\mathrm{avg}}}\right)*100$$

### Mean absolute percentage error

Mean Absolute Percentage Error (MAPE) is a measure of difference between two continuous variables, which is given by the Eq. (27).

27 $$\mathrm{MAPE}=\frac{\sum_{\mathrm{i}=1}^{\mathrm{n}}\left|\frac{{\mathrm{S}}_{\mathrm{i}}-{\mathrm{O}}_{\mathrm{i}}}{{\mathrm{O}}_{\mathrm{i}}}\right|\times 100}{\mathrm{n}}$$

MAPE is less sensitive to extreme values and therefore, it is intuitively more appealing than RMSE. However, for evaluating the performance of model both RMSE and MAPE can be used as many researchers employed the both indices^50,57^.

## Supplementary Information

Supplementary Information.

## Data Availability

The data used for the study are available from the corresponding author on reasonable request.
