# Supplementary material for: Identifying appropriate prediction models for estimating hourly temperature over diverse agro-ecological regions of India
Source: Sci Rep. 2023 May 13;13:7789. doi: 10.1038/s41598-023-34194-9 (PMC10183030; doi:10.1038/s41598-023-34194-9)
Supplement: Supplementary file 1 — Supplementary Information. [file 41598_2023_34194_MOESM1_ESM.pdf]

Title Page

Identifying appropriate prediction models for estimating hourly temperature over diverse agro-ecological regions of India

Santanu Kumar Bal, V.P. Pramod, V.M. Sandeep, N. Manikandan*, M.A. Sarath Chandran, A.V.M. Subba Rao, P. Vijaya Kumar, M. Vanaja and V.K. Singh

Santanu Kumar Bal, ICAR-Central Research Institute for Dryland Agriculture, Hyderabad, Telangana, INDIA 500059

V.P. Pramod, ICAR-Central Research Institute for Dryland Agriculture, Hyderabad, Telangana, INDIA 500059

V.M. Sandeep, ICAR-Central Research Institute for Dryland Agriculture, Hyderabad, Telangana, INDIA 500059

N. Manikandan, ICAR-Central Research Institute for Dryland Agriculture, Hyderabad, Telangana, INDIA 500059

M.A. Sarath Chandran, ICAR-Central Research Institute for Dryland Agriculture, Hyderabad, Telangana, INDIA 500059

A.V.M. Subba Rao, ICAR-Central Research Institute for Dryland Agriculture, Hyderabad, Telangana, INDIA 500059

P. Vijaya Kumar, ICAR-Central Research Institute for Dryland Agriculture, Hyderabad, Telangana, INDIA 500059

M. Vanaja, ICAR-Central Research Institute for Dryland Agriculture, Hyderabad, Telangana, INDIA 500059

V.K. Singh, ICAR-Central Research Institute for Dryland Agriculture, Hyderabad, Telangana, INDIA 500059

***Corresponding author**

N. Manikandan, ICAR-Central Research Institute for Dryland Agriculture, Hyderabad, Telangana, INDIA 500059

E-mail: manikandan.narayanan@icar.gov.in

Supplementary Table

Table S1. Geographical details of the selected locations

S. No.	State	District	Location	Latitude	Longitude	Altitude	Physiography	Soils	Climate
1	Andhra Pradesh	Anantapur	Reddipalli	14.72	77.67	334	Deccan Plateau	Red & Black Soils	Hot Arid
2	Andhra Pradesh	Kurnool	Yagantipalli	15.32	78.21	233	Deccan Plateau	Red & Black Soils	Hot Semi-Arid
3	Andhra Pradesh	West Godavari	Pedavegi	16.81	81.13	49	Deccan Plateau	Red & Black Soils	Hot Semi-Arid
4	Assam	Golaghat	Khumtai	26.59	93.84	90	Assam & Bengal Plain	Alluvium Derived Soils	Hot Sub-humid to Humid
5	Bihar	Aurangabad	Aurangabad	24.70	84.35	122	Northern Plain	Alluvium Derived Soils	Hot Sub-humid
6	Bihar	Madhubani	Madhubani	26.34	86.07	57	Eastern Plain	Alluvium Derived Soils	Hot Sub-humid
7	Bihar	Nawada	Sokhodeora	24.88	85.54	88	Northern Plain	Alluvium Derived Soils	Hot Sub-humid
8	Chhattisgarh	Bilaspur	Bilaspur	22.09	82.14	258	Eastern Plateau	Red & Yellow Soils	Hot Sub-humid
9	Chhattisgarh	Raipur	Bhatapara	21.23	81.70	289	Eastern Plateau	Red & Yellow Soils	Hot Sub-humid
10	Himachal Pradesh	Hamirpur	Bara	31.68	76.52	774	Western Himalayas	Brown Forest and Podzolic Soils	Warm Sub-humid
11	Himachal Pradesh	Kangra	Kangra	32.09	76.26	784	Western Himalayas	Brown Forest and Podzolic Soils	Warm Sub-humid
12	Himachal Pradesh	Mandi	Sundernagar	31.59	76.91	800	Western Himalayas	Brown Forest and Podzolic Soils	Warm Sub-humid
13	Himachal Pradesh	Kinnaur	Sangla	31.42	78.26	2642	Western Himalayas	Brown Forest and Podzolic Soils	Warm Sub-humid
14	Himachal Pradesh	Sirmaur	Dhaulakuan	30.56	77.47	1008	Western Himalayas	Brown Forest and Podzolic Soils	Warm Sub-humid

S. No.	State	District	Location	Latitude	Longitude	Altitude	Physiography	Soils	Climate
15	Karnataka	Belgaum	Tukkanatti	15.84	74.49	773	Deccan Plateau	Shallow & Medium Black Soils	Hot Semi-Arid
16	Karnataka	Chintamani	Chintamani	13.34	78.08	866	Eastern Ghats & TN Uplands	Red Loamy Soils	Hot Semi-Arid
17	Kerala	Alleppy	Kayamkulam	9.14	76.51	8	Western Ghats	Red & Lateritic Soils	Hot Humid-Per-humid
18	Kerala	Thrissur	Kannara	10.53	76.32	18	Western Ghats	Red & Lateritic Soils	Hot Humid-Per-humid
19	Madhya Pradesh	Chhatarpur	Nowgaon	25.03	79.46	248	Central Highlands	Red & Black Soils	Hot Semi-Arid
20	Madhya Pradesh	Satna	Majhgawan	24.60	80.83	326	Central Highlands	Red & Black Soils	Hot Sub-humid
21	Maharashtra	Aurangabad	Aurangabad	19.85	75.57	697	Deccan Plateau	Shallow & Medium Black Soils	Hot Semi-Arid
22	Maharashtra	Nandurbar	Kolde	21.43	74.28	156	Deccan Plateau	Shallow & Medium Black Soils	Hot Semi-Arid
23	Maharashtra	Pune	NIASM	18.15	74.50	575	Deccan Plateau	Shallow & Medium Black Soils	Hot Semi-Arid
24	Maharashtra	Pune	Baramati	18.14	74.52	559	Deccan Plateau	Shallow & Medium Black Soils	Hot Semi-Arid
25	Maharashtra	Gondia	Hiwara	21.47	80.18	309	Central Highlands	Red & Black Soils	Hot Sub-humid
26	Mizoram	Lunglei	Hnathial	22.95	92.93	887	North Eastern Hills	Red & Lateritic Soils	Warm Per-humid
27	Odisha	Ganjam	Ganjam	19.38	85.05	7	Eastern Coastal Plain	Coastal Alluvium Derived Soils	Hot Sub-humid to Semi-Arid
28	Odisha	Jharsugda	Jharsugda	21.85	84.00	224	Eastern Plateau	Red & Lateritic Soils	Hot Sub-humid
29	Odisha	Kendrapara	Kapaleswar	20.52	86.44	6	Eastern Coastal Plain	Coastal Alluvium Derived Soils	Hot Sub-humid to Semi-Arid

S. No.	State	District	Location	Latitude	Longitude	Altitude	Physiography	Soils	Climate
30	Odisha	Sonepur	Sonepur	20.84	83.89	119	Eastern Plateau	Red & Lateritic Soils	Hot Sub-humid
31	Rajasthan	Udaipur	Udaipur	24.60	73.70	576	Northern Plain	Alluvium Derived Soils	Hot Semi-Arid
32	Sikkim	East Sikkim	Ranipool	27.28	88.59	814	Eastern Himalayas	Brown and Red Hill Soils	Warm Per-humid
33	Tamil Nadu	Namakkal	Namakkal	11.21	78.16	196	Eastern Ghats & TN Uplands	Red Loamy Soils	Hot Semi-Arid
34	Tamil Nadu	Ramanathapuram	Ramanathapuram	9.36	78.83	9	Eastern Coastal Plain	Coastal Alluvium Derived Soils	Hot Sub-humid to Semi-Arid
35	Tamil Nadu	Villupuram	Thindivanam	11.94	79.48	46	Eastern Ghats & TN Uplands	Red Loamy Soils	Hot Semi-Arid
36	Telangana	Nalagonda	Gaddipalli	16.85	79.45	150	Deccan Plateau	Red & Black Soils	Hot Semi-Arid
37	Telangana	Ranga Reddi	Hayathnagar	17.34	78.58	519	Deccan Plateau	Red & Black Soils	Hot Semi-Arid
38	Uttar Pradesh	Baghpat	Baghpat	28.87	77.25	216	Northern Plain	Alluvium Derived Soils	Hot Semi-Arid
39	Uttar Pradesh	Bahraich	Bahraich	27.56	81.59	117	Eastern Plain	Alluvium Derived Soils	Hot Sub-humid
40	Uttar Pradesh	Gonda	Gopalpur	27.13	81.96	108	Eastern Plain	Alluvium Derived Soils	Hot Sub-humid
41	Uttar Pradesh	Gorakhpur	Belipur	26.76	83.37	78	Eastern Plain	Alluvium Derived Soils	Hot Sub-humid
42	Uttar Pradesh	Kushinagar	Kushinagar	26.73	83.88	79	Eastern Plain	Alluvium Derived Soils	Hot Sub-humid
43	Uttar Pradesh	Maharajganj	Maharajganj	27.16	83.57	91	Eastern Plain	Alluvium Derived Soils	Hot Sub-humid
44	Uttarakhand	Uttarkashi	Chinyalisaur	30.57	78.32	850	Western Himalayas	Brown Forest and Podzolic Soils	Warm Sub-humid

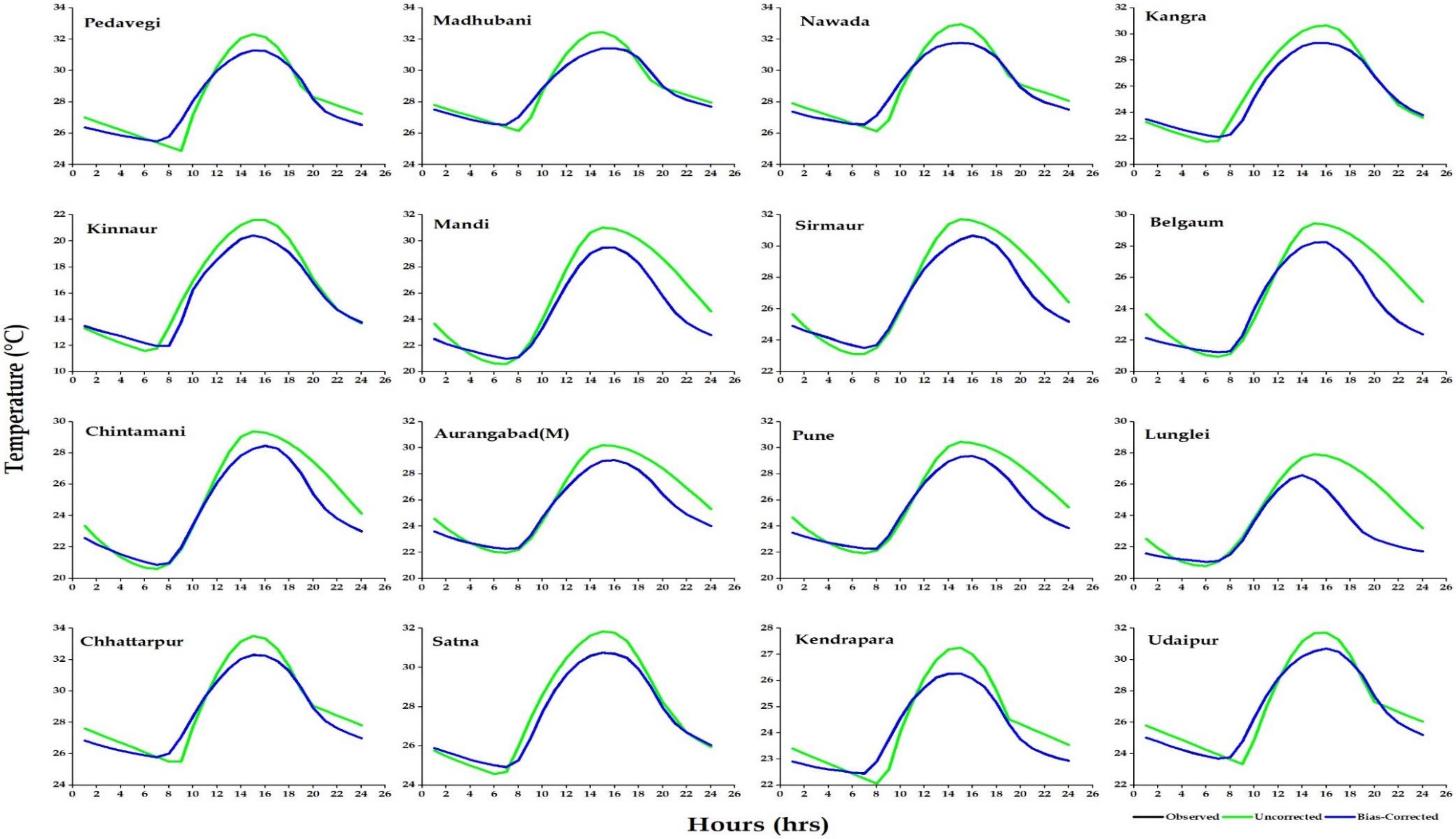

Fig. S1 Continued

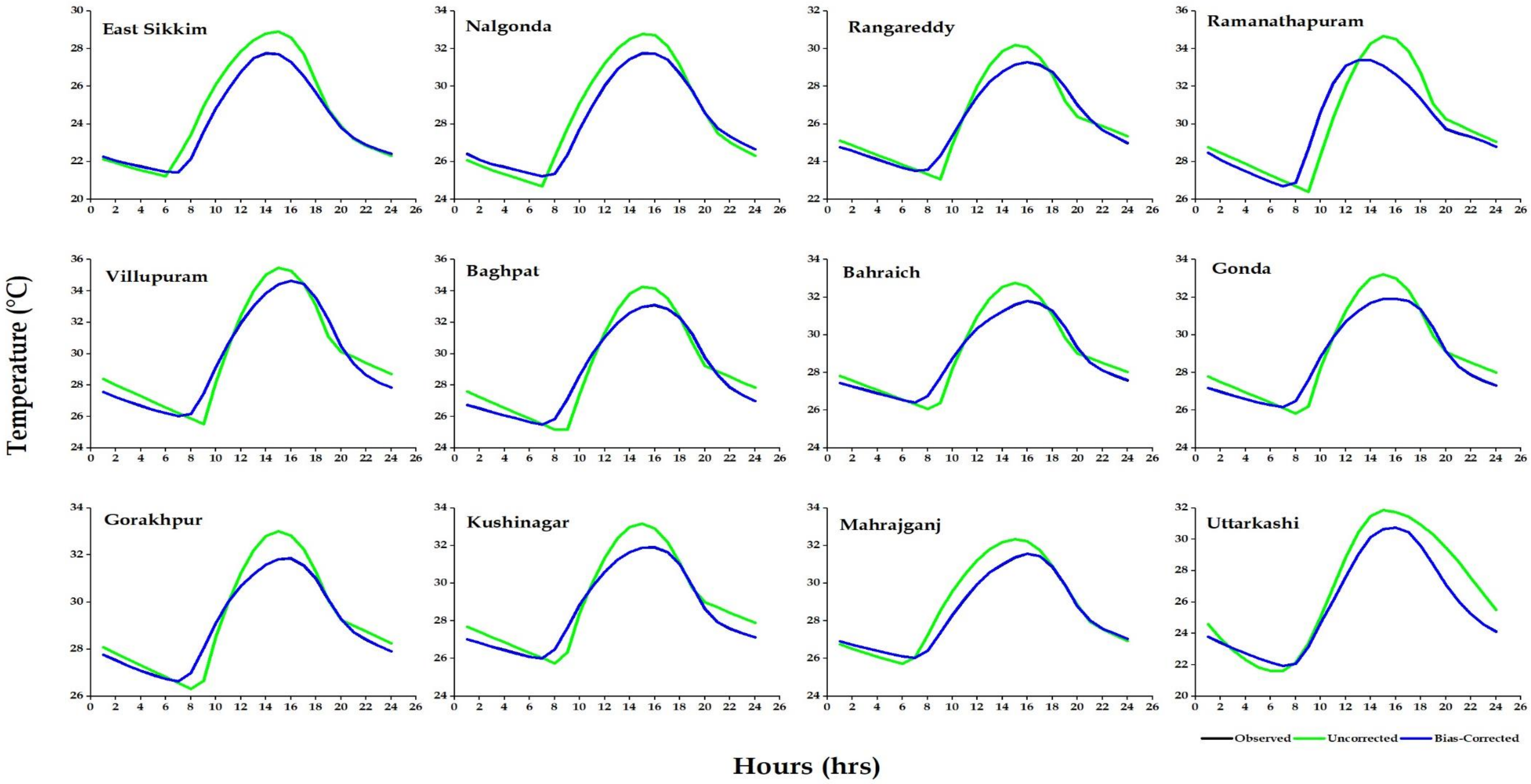

Figure S1. Line plots of observed, uncorrected and bias-corrected hourly air temperature during the *kharif* season (Lines of observed and bias-corrected are overlapped)

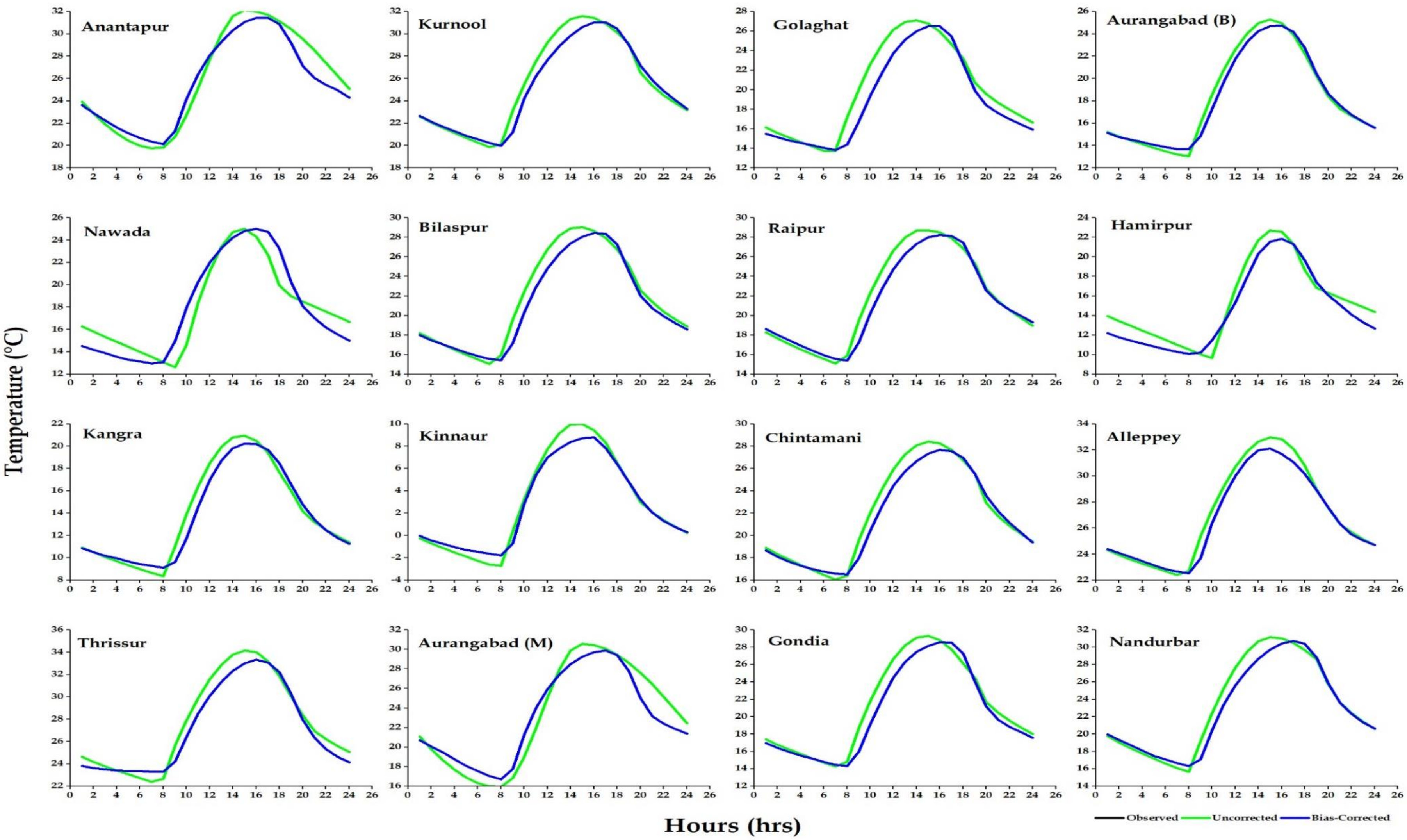

Fig. S2 Continued

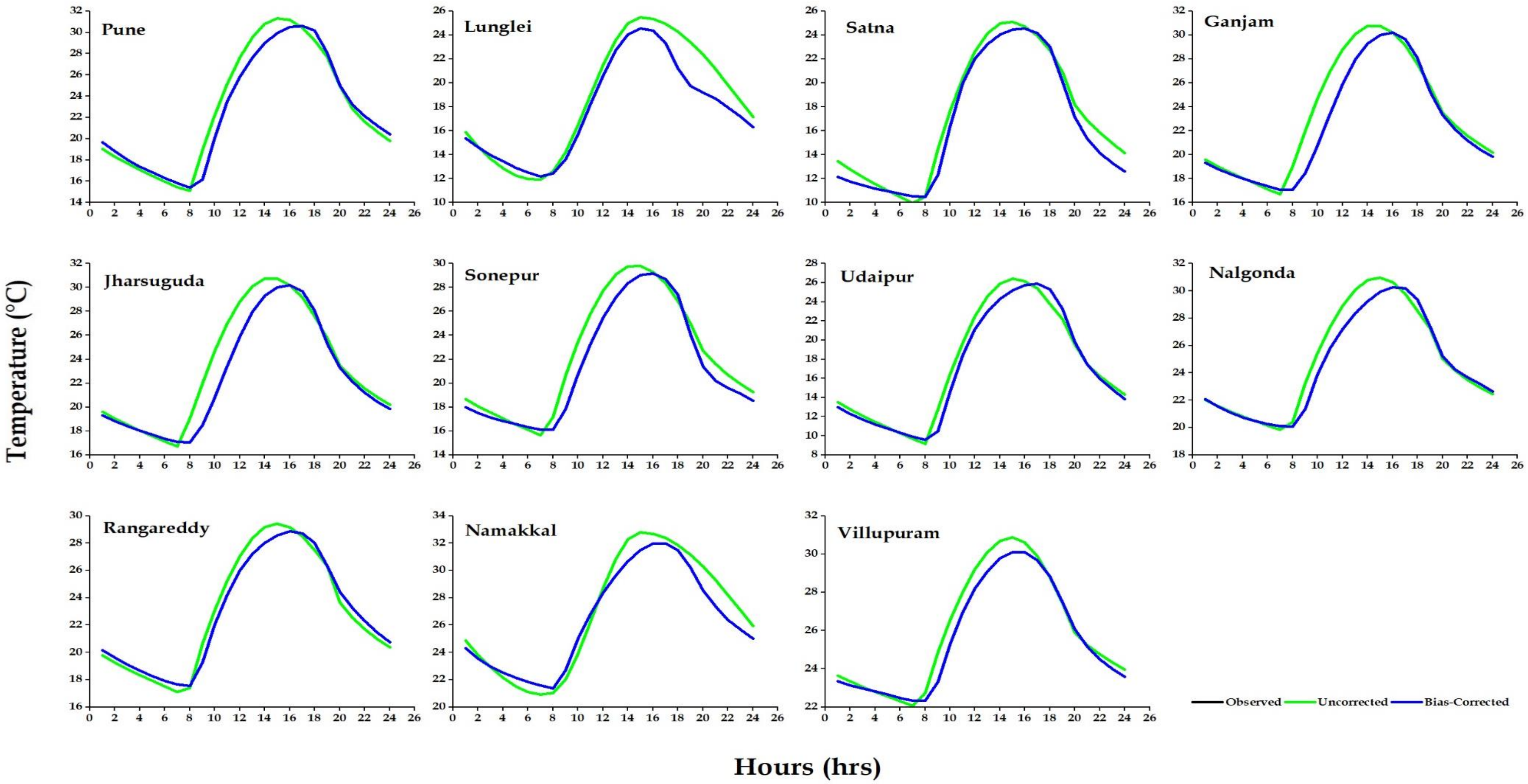

Figure S2. Line plots of observed, uncorrected and bias-corrected hourly air temperature during the *rabi* season (Lines of observed and bias-corrected are overlapped)
